# The effect of intra-cerebroventricular injection of insulin on the levels of monoamines on the raphe magnus nucleus of non-diabetic and short-term diabetic rats in the formalin test

**DOI:** 10.22038/ijbms.2019.35580.8485

**Published:** 2019-08

**Authors:** Shima Balali Dehkordi, Javad Sajedianfard, Ali Akbar Owji

**Affiliations:** 1 Department of Basic Sciences, School of Veterinary Medicine, Shiraz University, Shiraz, Iran; 2 Department of Biochemistry, School of Medicine, Shiraz University of Medical Sciences, Shiraz, Iran

**Keywords:** Formalin test, Insulin, Microdialysis, Monoamines, Pain

## Abstract

**Objective(s):**

Systemic and intracerebroventricular (ICV) injection of insulin possess analgesic effects. The raphe magnus nucleus (RMN) is part of the endogenous analgesia system. The objective of the present study was to evaluate the effects of ICV injection of insulin on the levels of monoamines and their related metabolites in the RMN during the formalin test in non-diabetic and short-term diabetic rats.

**Materials and Methods:**

Sixty four adult male rats were used. Diabetes was induced by Streptozotocin (STZ) (60 mg/kg, IP); insulin (5 mU/animal, 5 μl) was injected into the left ventricle. Microdialysis was performed in each rat. Samples were collected at 15 min intervals. After taking the base sample of microdialysis, 50 μl of 2.5% formalin was injected into the plantar surface of the hind paw, and the level of nociception was recorded every 15 sec for 1 hr. Monoamines and their metabolites concentrations were measured using the HPLC-ECD method.

**Results:**

Findings showed that ICV injection of insulin in non-diabetic rats increased the concentration of monoamines and their related metabolites in the RMN. In diabetic rats, injection of insulin decreased the concentrations of monoamines and their related metabolites in the RMN (*P*<0.05). Our results determined that, at least in part, insulin is associated with antinociceptive effect in non-diabetic rats.

**Conclusion:**

Based on the results, it seems that ICV injection of insulin in non-diabetic rats increased the activity of the central pain control pathways leading to antinociceptive response, but this condition was not seen in diabetic rats.

## Introduction

Pain pathways in the central nervous system (CNS) are complex and controlled by various mediators (1, 2, 3). Pain is controlled by certain pathways in the CNS. One of these paths is the descending serotonergic system originating from the nucleus raphe magnus (NRM) (3). The main function of NRM is pain mediation via sending projections to the dorsal horn of the spinal cord, which directly inhibits pain (4).

 NRM is a subset of raphe nuclei collections (2, 5). NRM is located in the caudal pons and medulla, which receives descending afferents from the periaqueductal grey matter (PAG), the paraventricular hypothalamic nucleus, central nucleus of the amygdala, lateral hypothalamic area, parvocellular reticular nucleus, the prelimbic, infralimbic, and medial and lateral precentral cortices (2, 5). It has been documented that NRM receives noradrenergic projections from the locus coeruleus nucleus (6); and proposed that some factors can alter the pain threshold including gender, depression, individual differences, and endocrine hormones (7).

Insulin is produced by the beta cells of the pancreas (8). Streptozotocin (STZ) is a glucosamine-nitrosourea compound that is used to induce diabetes by destroying pancreatic beta cells (9).

Insulin could cross the blood-brain barrier (BBB) via a receptor-mediated transport mechanism (10-13). Previous studies have shown that there are insulin receptors throughout the brain (11, 14). There are several lines of evidence indicating that insulin is synthesized in the brain especially in the hippocampus, olfactory bulb, piriform cortex, and the Purkinje cells of the cerebellar cortex (15, 16).

It has been found that insulin reduces pain sensitivity in rats (17, 18). In our previous study, we demonstrated that intracerebroventricular (ICV) injection of insulin in diabetic rats increased pain sensitivity while in non-diabetic rats, it increased the pain threshold (19).

Despite the studies above, there is no information on the central analgesic mechanism of insulin. The NRM is part of the endogenous analgesia system and operated in the spinal cord to control pain (20). Measurement of NRM neurotransmitters by microdialysis is considered a valid method for evaluation of NRM function (21). So the objective of this study was to evaluate the effect of ICV insulin injection on the concentration of neurotransmitters such as serotonin, dopamine, norepinephrine, and their metabolites in the NRM during formalin test in non-diabetic and short- term diabetic rats.

## Materials and Methods

***Animals***

Ethics: The protocol of this study was in accordance with the Ethics Committee of the School of Veterinary Medicine, Shiraz University, Shiraz, Iran (ethics committee code 93GCU3M1293).

Sixty-four male Sprague-Dawley adult rats (280 ± 30 g) were divided into eight groups (4 non–diabetic groups and 4 diabetic groups).

Animals were housed in standard Plexiglas boxes under a 12-hour light/dark cycle, with an ambient temperature of 22 ±2 ^°^C. They had free access to food and water.

***Study design***

The animals were divided into eight groups (n=8): group 1 (non-diabetic main control group) injected with normal saline (5 μl, ICV and 50 μl subcutaneously (SC) in the left hind paw); group 2 (non-diabetic control insulin group) injected with normal saline (5 μl, ICV) and formalin (50 μl, SC, in the left hind paw); group 3 (non-diabetic control formalin test group) injected with insulin (5 μl, ICV) and normal saline (50 μl, SC, in the left hind paw); group 4 (non-diabetic test group) injected with insulin (5 μl, ICV) and formalin (50 μl, SC, in the left hind paw); group 5 (diabetic control insulin group) injected with normal saline (5 μl, ICV) and formalin (50 μl, SC, in the left hind paw); group 6 (diabetic control formalin test group) injected with insulin (5 μl, ICV) and normal saline (50 μl, SC, in the left hind paw); group 7 (diabetic test group) injected with insulin (5 μl, ICV) and formalin (50 μl, SC, in the left hind paw); and group 8 (diabetic main control group) injected with normal saline (5 μl, ICV and 50 μl, SC, in the left hind paw). 

***Induction of diabetes***

After 24 hr fasting, a single intraperitoneal (IP) injection of STZ (60 mg/kg) (Sigma Aldrich Company) that was dissolved in 0.01 mol/l citric acid solution (pH=4.5) was used to induce diabetes (22, 23). Non-diabetic rats received an equal volume of citrate buffer (pH=4.5). Three days after the STZ administration, the blood glucose levels from the tail vein were measured by a glucometer (ACCU – CHECK). Blood glucose higher than 250 mg/dl was considered as a diabetic state (22, 23). Forty-eight hours after establishing the diabetes, the formalin test and microdialysis were performed.

***Preparation and calibration of the dialysis probe***

Concentric microdialysis probes with active dialysis length of 1 mm were made based on Sharp and Zetterstrom’s method (24). The dialysis membrane with spectra/pro hollow fiber: molecular weight cutoff: 6000 Da; 0.250 mm OD.

The probes were tested *in vitro* before being implanted in the rats’ brain. In this way, the ability of probes for recovering monoamines was determined (25). Recovery was about 25%.

***Probe and guide cannula implantation***

Rats were anesthetized by the intraperitoneal injection of sodium pentobarbital (50 mg/kg). The guide cannula was implanted in the lateral ventricle (AP=- 0.8 mm, L=+1.5 mm and DV=-3.6 mm) (26). The microdialysis probe was implanted in NRM (AP=-10.8 mm, L=0 mm, DV=-10.6 mm). Microdialysis and formalin test were done 24 hr after the implantation of probe and guide cannula (27, 28).

***Artificial cerebrospinal fluid (ACSF) and insulin preparation***

The components of ACSF (in mmol) were included: NaCl 114, NaOH 1, CaCl_2_ 1, MgSO_4_ 2, NaH_2_PO_4_ 1.25, KCl 3, NaHCO_3_ 26, Glucose 10 and pH= 7.4.

Insulin (bovine insulin) (Sigma Aldrich, Germany) was dissolved in saline and injected intracerebroventricularly.

***Microdialysis and formalin test***

Microdialysis was done 24 hr after stereotaxic surgery (27, 28). ACSF was continuously perfused into the microdialysis probe with a flow rate of 2 μl/min by a syringe pump (WPI, SP 210). Samples were collected at 15 min intervals. The perfused fluid was collected in microtubes located in ice. Insulin (5 mU/animal, 5 μl) was injected into the left ventricle at the rate of 1 μl /min by a Hamilton syringe (10 μl) in diabetic rats. Other animals received normal saline. Two base samples were collected (S1= without insulin effect, S2= with insulin effect). The formalin test was performed 10 min after the insulin injection. Dialysis samples were collected during the formalin test. Samples were stored at -80 ^°^C until HPLC analysis.

To perform the formalin test, 50 μl of 2.5% formalin was injected subcutaneously into the plantar region of the left hind paw using a 27-gauge needle. Normal saline was injected instead of formalin in related groups. The level of nociception was recorded every 15 sec for one hour (29). In the formalin test, the mean of pain scores during the early phase (first 5 min), and the late phase (20–60 min) were recorded.

***Histological verification***

**Figure 1:**
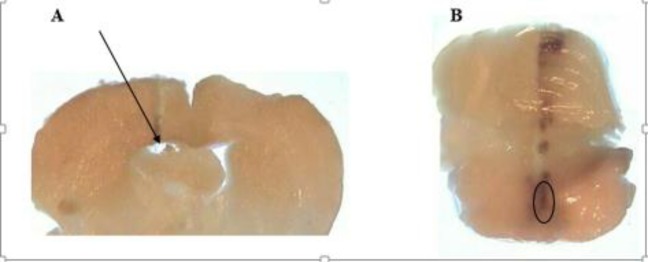
A: Example of histological verification of the cannula placement for microinjection of insulin/normal saline into the lateral ventricle. B: Example of histological verification of the dialysis probe placement in NRM

At the end of the microdialysis experiment, each animal was euthanized with an overdose of diethyl ether. Brains were removed and placed in formalin; then, histologic sections were provided (Figure 1). The position of guide cannula and probe tracing were verified according to rat brain atlas (26).

***High-pressure liquid chromatography (HPLC) analysis***

Norepinephrine, serotonin, dopamine, and their metabolites were analyzed using HPLC with electrochemical detection (ECD). Dialysate samples were injected into the column (Reverse-phase column, Eurospher, 100- 5 C18, 250× 4.6mm) connected to a pump (Knauer) and the electrochemical detector (Amperometric detector EC 3000). Autochro data module software was used for drawing the graph and data analysis. The oxidizing potential of the working electrode was set at +750 mV versus Ag| Ag Cl reference electrode.

The mobile phase composition contained 1-octane-sulfonic acid 360 mg, sodium phosphate 8.4 g, EDTA 30 mg and 16% of methanol in 1000 ml H_2_O (pH=3.5). The flow rate of mobile phase was 1.0 ml/min.

***Data analysis***

The Statistic analysis was done by SPSS 16.0 software. Two-way repeated measure ANOVA was used for the analysis. Results are expressed as mean±SEM. *P*<0.05 was considered as statistical significance.

## Results

***Microdialysis results***

**Figure 2:**
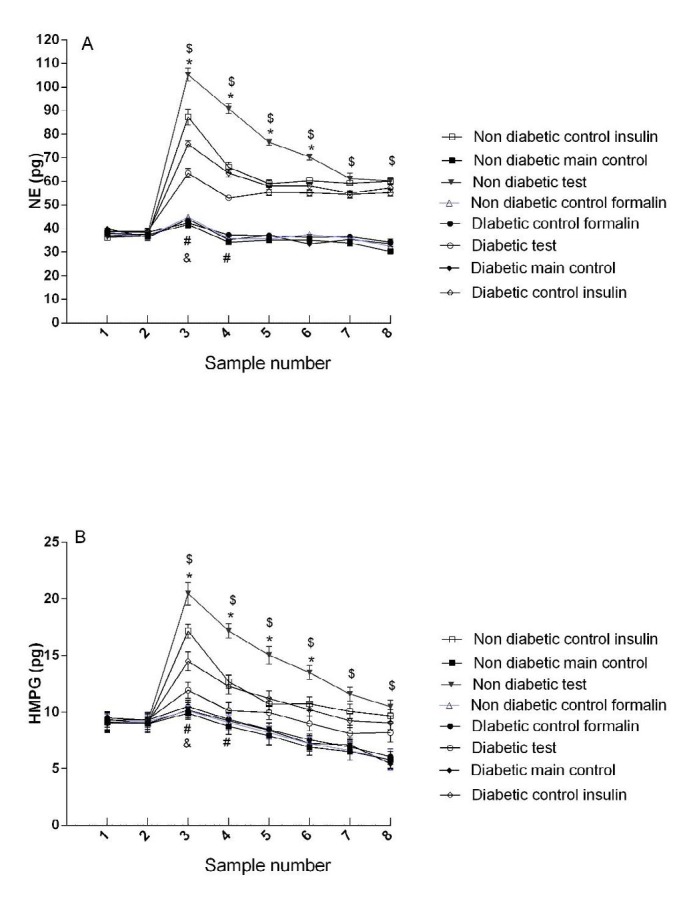
Concentration of norepinephrine (NE) and its metabolite (HMPG) in diabetic and non-diabetic groups. Sample numbers are according to collection of microdialysis samples every 15 min

**Figure 3:**
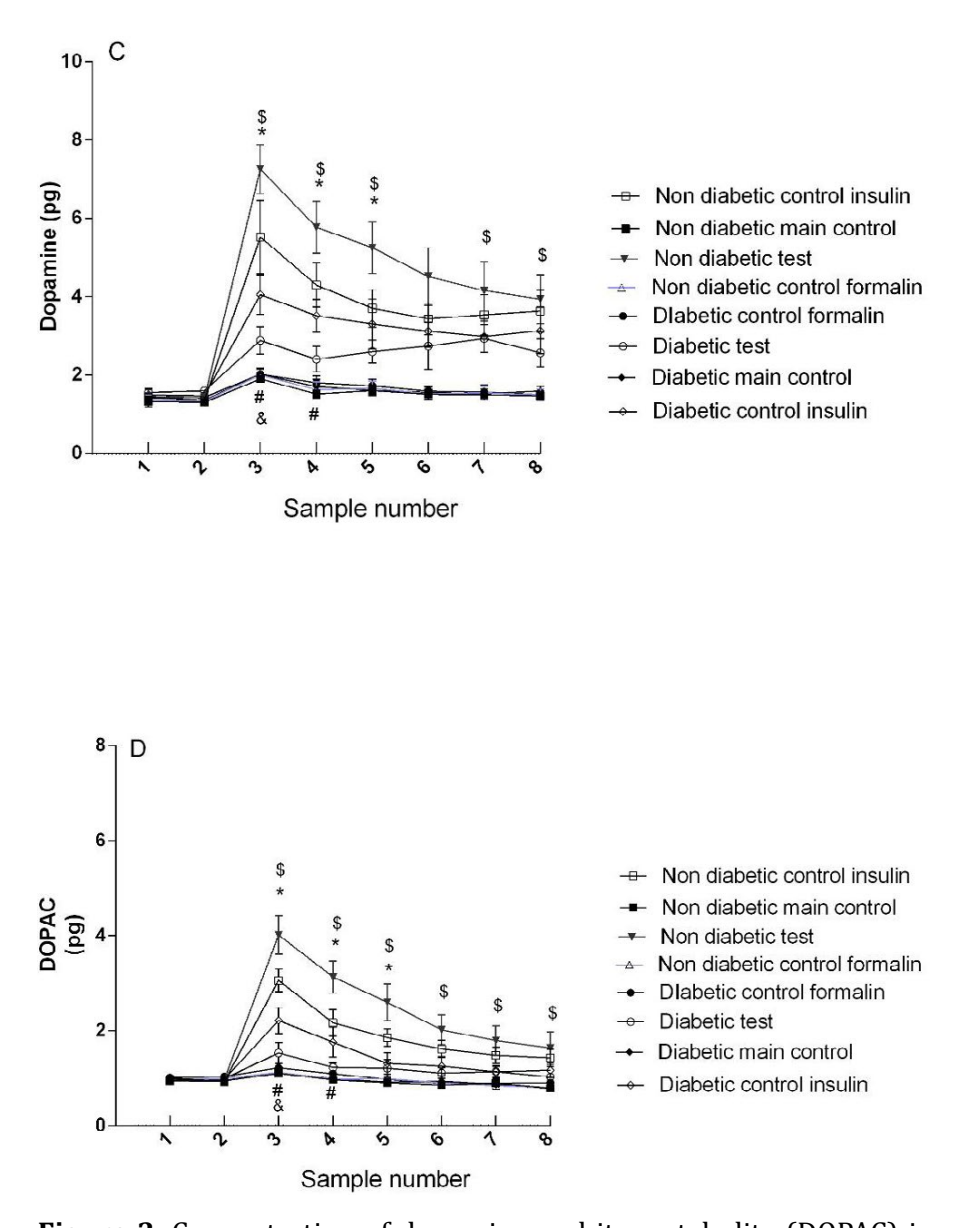
Concentration of dopamine and its metabolite (DOPAC) in diabetic and non-diabetic groups. Sample numbers are according to collection of microdialysis samples every 15 min

**Figure 4:**
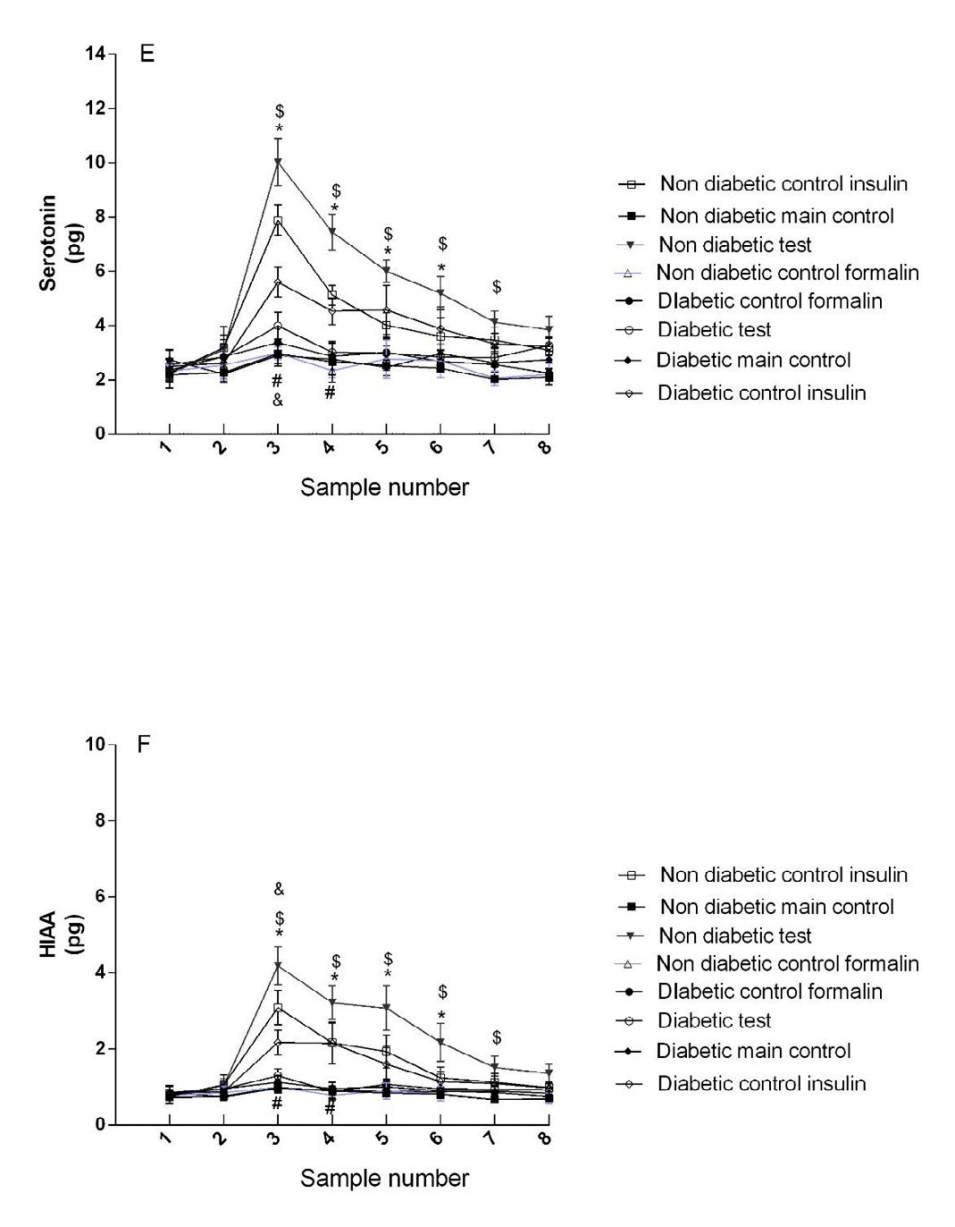
Concentration of serotonin and its metabolite (HIAA) in diabetic and non-diabetic groups. Sample numbers are according to collection of microdialysis samples every 15 min

The mean± SEM concentration of norepinephrine and its metabolite (MHPG), serotonin and its metabolite (HIAA) and dopamine and its metabolite (DOPAC) in each group are illustrated in Figures 2, 3 and 4.

***ICV injection of insulin increased the concentration of norepinephrine in the non-diabetic rats***

There was no significant difference between the non-diabetic control groups (group 1 with 3). 

The norepinephrine concentration was higher in the non-diabetic main test group (group 4) than in non-diabetic control insulin group (group 2). A significant difference was observed between these groups in the third, fourth, fifth, and sixth samples (*P*<0.05) (Figure 2A). There was no significant difference between diabetic control groups (groups 6 and 8) (Figure 2A).

The norepinephrine concentration was higher in the diabetic control insulin group (group 5) than in diabetic main test group (group 7). A significant difference was observed between these groups in the third and fourth samples (*P*<0.05) (Figure 2A). Our findings showed a significant difference between non-diabetic and diabetic main test groups (4 and 7 groups) in all samples except for the first and second base samples (Figure 2A).

There was significant difference between diabetic and non-diabetic control insulin groups in the first phase of the formalin test (5 and 2 groups) (F (37.30, 229.14) = 48.58, *P*<0.05).

***ICV injection of insulin increased the concentration of MHPG (3-Methoxy-4-hydroxyphenylglycol) in the non-diabetic rats***

There was no significant difference between non-diabetic control groups (groups 1 and 3) (Figure 2B).

MHPG concentration was higher in the non-diabetic main test group (group 4) than in non-diabetic control insulin group (group 2). A significant difference was observed between these groups in the third, fourth, fifth, and sixth samples (*P*<0.05) (Figure 2B). 

There was no significant difference between diabetic control groups (groups 6 and 8) (Figure 2B).

MHPG concentration was higher in the diabetic control insulin group (group 5) than in diabetic main test group (group 7). A significant difference was observed between these groups in the third and fourth samples (*P*<0.05) (Figure 2B). 

There was a significant difference between non-diabetic and diabetic main test groups (4 and 7 groups) in all samples except for the first and second base samples (*P*<0.05) (Figure 2B).

There was significant difference between diabetic and non-diabetic control insulin groups in the first phase of the formalin test (5 and 2 groups) (F (20.12, 123.60) = 9.55, *P*<0.05).

***ICV injection of insulin increased the concentration of dopamine in the non-diabetic rats***

There was no significant difference between non-diabetic control groups (group 1 and 3). 

The dopamine concentration was higher in the non-diabetic main test group (group 4) than in non-diabetic control insulin group (group 2). A significant difference was observed between these groups in the third, fourth, and fifth samples (*P*<0.05) (Figure 3C). 

There was no significant difference between diabetic control groups (group 6 and 8) (Figure 3C).

The dopamine concentration was higher in the diabetic control insulin group (group 5) than in diabetic main test group (group 7). A significant difference was observed between these groups in the third and fourth samples (*P*<0.05) (Figure 3C). 

There was a significant difference between non-diabetic and diabetic main test groups (4 and 7 groups) in all samples except for the first and second base samples and the sixth sample (Figure 3C).

There was significant difference between diabetic and non-diabetic control insulin groups in the first phase of the formalin test (groups 5 and 2) (F (24.96, 153.31) = 5.58, *P*<0.05).

***ICV injection of insulin increased the concentration of DOPAC (3, 4 hydroxyphenyl acetaldehyde) in the non-diabetic rats***

There was no significant difference between non-diabetic control groups (group 1 and 3). 

The DOPAC concentration was higher in the non-diabetic main test group (group 4) than in non-diabetic control insulin group (group 2). A significant difference was observed between these groups in the third, fourth, and fifth samples (*P*<0.05) (Figure 3D). 

There was no significant difference between diabetic control groups (group 6 and 8) (Figure 3D).

The DOPAC concentration was higher in diabetic control insulin group (group 5) than in diabetic main test group (group 7). A significant difference was observed between these groups in the third and fourth samples (*P*<0.05) (Figure 3D). 

There was a significant difference between non-diabetic and diabetic main test groups (4 and 7 groups) in all samples except for the first and second base samples (Figure 3D).

There was a significant difference between diabetic and non-diabetic control insulin groups in the first phase of the formalin test (5 and 2 groups) (F (22.92, 140.78) = 12.92, *P*<0.05).

***ICV injection of insulin increased the concentration of serotonin in the non-diabetic rats***

There was no significant difference between non-diabetic control groups (groups 1 and 3) (Figure 4E).

The serotonin concentration was higher in the non-diabetic main test group (group 4) than in non-diabetic control insulin group (group 2). A significant difference was observed between these groups in the third, fourth, fifth, and sixth samples (*P*<0.05) (Figure 4E). 

There was no significant difference between diabetic control groups (groups 6 and 8) (Figure 4E).

The serotonin concentration was higher in the diabetic control insulin group (group 5) than in diabetic main test group (group 7). A significant difference was observed between these groups in the third and fourth samples (*P*<0.05) (Figure 4E).

There was a significant difference between the non-diabetic and diabetic main test groups (4 and 7 groups) in all samples except for the first, and second base and eighth samples (*P*<0.05) (Figure 4E).

There was a significant difference between diabetic and non-diabetic control insulin groups in the first phase of the formalin test (groups 5 and 2) (F (49, 301) = 4.84, *P*<0.05).

* A significant difference between non-diabetic control insulin and test groups (*P*<0.05)

$ A significant difference between non-diabetic and diabetic test groups (*P*<0.05)

# A significant difference between diabetic control insulin and test groups (*P*< 0.05)

& A significant difference between non-diabetic and diabetic control insulin groups (*P*<0.05)

* A significant difference between non-diabetic control insulin and test groups (*P*<0.05)

$ A significant difference between non-diabetic and diabetic test groups (*P*<0.05)

# A significant difference between diabetic control insulin and test groups (*P*<0.05)

& A significant difference between non-diabetic and diabetic control insulin groups (*P*<0.05)

* A significant difference between non-diabetic control insulin and test groups (*P*< 0.05)

$ A significant difference between non-diabetic and diabetic test groups (*P*<0.05)

# A significant difference between diabetic control insulin and test groups (*P*<0.05)

& A significant difference between non-diabetic and diabetic control insulin groups (*P*< 0.05)

***ICV injection of insulin increased the concentration of 5- HIAA (5-hydroxyindoleacetic Acid) in the non-diabetic rats***

There was no significant difference between the non-diabetic control groups (groups 1 and 3) (Figure 4F).

The HIAA concentration was higher in the non-diabetic main test group (group 4) than in non-diabetic control insulin group (group 2). A significant difference was observed between these groups in the third, fourth, fifth, and sixth samples (*P*<0.05) (Figure 4F). 

There was no significant difference between diabetic control groups (groups 6 and 8) (Figure 4F).

The HIAA concentration was higher in diabetic control insulin group (group 5) than in diabetic main test group (group 7). A significant difference was observed between these groups in the third and fourth samples (*P*<0.05) (Figure 4F). 

There was a significant difference between the non-diabetic and diabetic main test groups (4 and 7 groups) in all samples except for the first, and second base and eighth samples (*P*<0.05) (Figure 4F).

There was a significant difference between the diabetic and non-diabetic control insulin groups in the first phase of the formalin test (5 and 2 groups) (F (29.58, 181.71) = 4.21, *P*<0.05).

## Discussion

Several studies have suggested an analgesic effect for insulin (17, 18, 30, 31). It has been proposed that insulin exert its analgesic effects through affecting the analgesic pathways, including dopaminergic, serotonergic, and opioidergic systems (18). We recently reported that ICV injection of insulin exerted an analgesic effect on rats. Moreover, we showed that ICV injection of insulin in diabetic rats could increase the pain responses in the formalin test (19).

Therefore, in the current study, we aimed to clarify the mechanisms involved in this effect, focusing on norepinephrine, serotonin, dopamine, and their metabolites in the NRM in the formalin test. 

Our results demonstrated that the concentration of norepinephrine and its metabolite (MHPG), serotonin and its metabolite (5- HIAA), as well as dopamine and its metabolite (DOPAC) in the NRM, increased after the formalin-induced pain response. We showed that the concentration of the aforementioned neurotransmitters and also pain response in non-diabetic control insulin group, was higher than the short-term diabetic control insulin group. 

Peripheral nerves such as Aδ and C fibers conduct impulses to the lumbosacral cord and the paragigantocellularis in the rostroventral medulla (32). The stimulation of the paragigantocellularis increases the release of glutamate and aspartate in the locus coeruleus (LC), leading to the stimulation of the noradrenergic neurons within the locus coeruleus (33, 34). Norepinephrine activates the α_2_- adrenoceptors in the descending noradrenergic pathway of the spinal cord, (34, 35). NRM receives noradrenergic projections from LC (6). Therefore, it is anticipated that the stimulation of noradrenergic neurons within LC increases the norepinephrine and its metabolite concentrations in NRM. The body of evidence demonstrated that the stimulation of α_1_-adrenoceptors in the raphe magnus neurons led to the increases in the neuronal activity of NRM, resulting in inhibition of nociception (6, 36).

NRM is the origin of the serotonergic pathway modulating the transmission of noxious inputs at the spinal level (37). The periaqueductal gray and nucleus paragigantocellularis send serotonin projection to the raphe nucleus, playing a role in pain relief (38).

Mesencephalic raphe nuclei contain substance P, CCK, VIP, dopamine, and neurotensin (39- 42). There are numerous dopamine-containing cell bodies in the dorsal and median raphe nuclei (43). 

The antinociceptive action of dopamine is mediated by D2 receptors in the rats’ nucleus raphe Magnus (44). 

It has been reported that in the diabetic state, in response to cell hypoglycemia, endogenous opiates are released acutely along with ACTH (45). Raz *et al*. suggested that in diabetes, the pain threshold was maintained due to the compensatory secretion of endogenous opiates (45). Hyperglycemia in diabetes, changes the function of the hypothalamic-pituitary and endogenous opioid systems, leading to the acute release of opioids (46). It shows that diabetes increases the level of serotonin in the CNS (47) resulting in an increased pain threshold (48- 50).

The results of the present study showed that non-diabetic and diabetic control groups had high levels of norepinephrine, dopamine, and their metabolites in the NRM at the time of saline injection in the hind paw when compared after the formalin injection. These results are in line with those of Sajedianfard *et al*. (51). The increased levels of these neurotransmitters are due to the stress imposed by the injection. It is known that noradrenergic neurons in the locus coeruleus respond forcefully to certain types of stress (52). Fernstrom reported that stressful stimuli increased norepinephrine, serotonin, and dopamine synthesis as well as the turnover in the rats’ brain (53).

In this study, ICV injection of insulin ten minutes before the induction of pain increased the norepinephrine, MHPG, serotonin, 5- HIAA, dopamine, and DOPAC concentrations in the non-diabetic main test group (group 4) compared with the non-diabetic control insulin group (group 2); this is in agreement with the results of our previous study (19). Thus, ICV injection of insulin may indirectly activate noradrenergic, serotonergic, and dopaminergic descending pain control pathways, thereby relieving pain in non-diabetic rats. It was reported that serotonin microinjection into the NRM produced significant analgesic effects. (54). Previous studies proposed a central role for dopamine in modulating the pain perception and analgesia within supraspinal regions (55, 56). It has been suggested that painful symptoms of Parkinson’s disease may be due to the decrease in the dopamine levels (56).

Study showed that ICV injection of insulin through the activation of central dopaminergic, serotonergic, and opioidergic pathways attenuated specifically the second phase of formalin-induced nociception in non-diabetic mice (18). Serotonin, dopamine, opioidergic receptors, NMDA receptors, and potassium and calcium channels may have an important role in the analgesic effect of insulin (17).

In the present study, ICV injection of insulin ten minutes before the induction of pain, decreased the concentration of norepinephrine, MHPG, serotonin, 5- HIAA, dopamine, and DOPAC in the diabetic main test group (group 7) compared with the diabetic control insulin group (group 5). The reduction of norepinephrine, serotonin, dopamine, and their metabolites was consonant with the increasing pain response (19). Our previous study showed that ICV injection of insulin in diabetic rats could not reduce the pain response and, partially, decreased the pain threshold (especially 5 to 25 min after the formalin injection) (19). Researchers reported that the analgesic effect of insulin on diabetic mice was less than that observed in non-diabetic mice due to the distribution of pain control pathways such as dopaminergic (57), serotonergic, and opioidergic pathways (18). Researchers showed that although insulin improved the pain responses of STZ-diabetic rats, it did not alter the levels of norepinephrine and serotonin in the brain stem and spinal cord. They concluded that the anti-nociceptive effects of insulin were not mediated by the noradrenergic and serotoninergic systems (23). These differences may be related to the model of diabetes induction in our study in which we used a short-term model of diabetes with other previous studies. 

## Conclusion

In the present study, ICV injection of insulin increased the concentration of serotonin, norepinephrine, dopamine, and their metabolites in the NRM of non-diabetic rats. The concentrations of these neurotransmitters and their metabolites in the diabetic rats were decreased after ICV injection of insulin. 

Based on these results, it seems that ICV injection of insulin in non-diabetic rats, increased the activity of the central descending pain control pathways, but this condition was not seen in the diabetic rats.
